# Development and validation of the Alcohol Message Perceived Effectiveness Scale

**DOI:** 10.1038/s41598-023-28141-x

**Published:** 2023-01-18

**Authors:** Michelle I. Jongenelis, Catherine Drane, Penelope Hasking, Tanya Chikritzhs, Peter Miller, Gerard Hastings, Simone Pettigrew

**Affiliations:** 1 Melbourne Centre for Behaviour Change, Melbourne School of Psychological Sciences, The University of Melbourne, Parkville, VIC 3056 Australia; 2 Future of Work Institute, Curtin University, Bentley, Australia; 3 School of Population Health, Curtin University, Bentley, Australia; 4 National Drug Research Institute, Curtin University, Bentley, Australia; 5 Centre for Drug Use, Addictive and Anti-Social Behaviour Research, Deakin University, Geelong, Australia; 6 Institute for Social Marketing, University of Stirling, Stirling, UK; 7 The George Institute for Global Health, University of New South Wales, Sydney, Australia

**Keywords:** Diseases, Risk factors

## Abstract

To assist intervention developers assess the likely effectiveness of messages designed to encourage greater use of protective behavioral strategies, this study developed and tested the Alcohol Message Perceived Effectiveness Scale (AMPES). Recommendations from the message effectiveness literature were used to guide AMPES development. The resulting scale was administered in online surveys at two time points to Australian drinkers aged 18–70 years (3001 at Time 1 and 1749 at Time 2). An exploratory factor analysis identified the presence of two factors (‘effect perceptions’ and ‘message perceptions’) that accounted for 71% of the variance in scores. Internal consistency of scores was good for the overall scale (ω = 0.83) and ‘effect perceptions’ factor (ω = 0.85), but suboptimal for the ‘message perceptions’ factor (α = 0.60). Scores on both factors significantly predicted enactment of protective behavioral strategies. The AMPES appears to be an appropriate tool to assess perceived message effectiveness and assist in the development of public health messages designed to reduce alcohol consumption.

## Introduction

Health communication campaigns are widely used to influence health-related behaviors^1^. Evidence indicates that such campaigns have the potential to produce positive changes and prevent negative changes in health-related behaviors across various populations^2^, which is likely to be at least partially due to their ability to rapidly transmit messages to large audiences^1^. To ensure the outcomes of health communication campaigns are optimized, it is critical that messages featured in such campaigns are tested for effectiveness prior to campaign production, implementation, and dissemination^2,3^. This is especially important in the context of constrained public health budgets and the need to identify effective and ineffective messages before allocating scarce and costly resources to campaign implementation^4^.

To aid the message selection process, researchers and developers have turned to measures of perceived message effectiveness (PME), defined as target audience ratings of the likely persuasive impact of messages^5^. PME is a widely used indicator of the potential for messages to change attitudes and behavior^4,6–8^, yet there is ongoing debate about the extent to which it is diagnostic of actual message effectiveness (AME), defined as the ability of a message to produce the intended persuasive effects on attitudes, intentions, and/or behaviors^9^. Meta-analyses examining the relationship between PME and AME across a range of behaviors have produced mixed results, with Dillard et al.^8^ observing a corrected-for-attenuation correlation of 0.41 between PME and AME and O’Keefe^9^ finding that measures of PME and AME matched only 58% of the time, a value that was not statistically different from chance. Evidence from studies conducted in specific health-related domains suggests PME is a valid indicator of actual effectiveness both cross-sectionally and longitudinally^3,5,8,10,11^. These mixed findings may be at least partially due to differing conceptualizations of PME and the significant heterogeneity in the PME measures used across studies^6^.

Advancing the conceptualization, validity, and reliability of PME measures is critical to improving the precision of tools that assess the likely effectiveness of campaign messages, thus maximizing campaign efficacy^6^. In their systematic review of PME measures in the context of tobacco control campaigns, Noar et al.^6^ made several recommendations for research in this area and PME scale development. The present study aimed to extend prior work and adopt these recommendations by developing and psychometrically evaluating a scale—the Alcohol Message Perceived Effectiveness Scale (AMPES)—that could be used to assess the perceived effectiveness of messages promoting engagement in alcohol-related protective behavioral strategies (PBSs). PBSs are actions used by individuals in an attempt to reduce their alcohol consumption and/or related harm^12^. Evidence suggests that PBS enactment can lower levels of alcohol consumption^13,14^, with PBSs that involve drink counting, deciding not to exceed a certain number of drinks, and refusing an unwanted alcohol beverage found in previous research to be associated with reductions in alcohol intake^15,16^.

Despite the potential benefits associated with regular use of PBSs^13,14^, enactment is generally low. For example, although national drinking guidelines are expressed in terms of quantity of standard drinks consumed, only half of Australian drinkers report counting their drinks most or all of the time^17^. There is thus an opportunity to develop health communication campaigns that promote increased use of PBSs, especially those that have been found to be most effective in reducing alcohol intake. In addition, the vast majority of work on PME has been conducted in the context of tobacco control, and there is a need to extend its application to other health behaviors^5^. The present study aimed to address these issues by examining the utility of a PME measure developed in the context of testing evidence-based protective behavioral strategies designed to reduce alcohol consumption.

## Method

### Scale development

As PME has been widely applied in tobacco education campaigns, a review of this literature was conducted to inform scale development. Six items with demonstrated ability to accurately predict intentions and behaviors^5,10,18,19^ were selected for inclusion in a scale relevant to PBS messages. Efforts were made to ensure the AMPES followed the recommendations outlined in Noar et al.’s^6^ systematic review and conceptualization of PME. Specifically, (i) the scale assessed both message and effect perceptions to allow for a comparison of the effectiveness of each perception type, (ii) the scale featured items that assessed a variety of persuasive constructs (e.g., personal relevance, motivation to act), (iii) scale items referred to the behavior being assessed (i.e., alcohol consumption), and (iv) a personal referent was used to improve measurement precision (i.e., items were phrased in the context of the individual’s own beliefs and behaviors). Items are presented in Table 1. Example items include “makes me stop and think about my drinking”, “motivates me to reduce the amount of alcohol I drink”, and “is easy to understand”.

**Table 1:** Item-level descriptive statistics, inter-item correlations, and factor loadings (*n* = 3001).

Please indicate the extent to which you agree/disagree that this alcohol message…	Mean (*SD*)	Skew	Kurt	1	2	3	4	5	6	Loadings (1)	Loadings (2)
Factor 1—effect perceptions											
1. …makes me stop and think about my drinking	3.28 (1.07)	− 0.37	− 0.60	–	0.56***	0.64***	0.58***	0.13***	0.31***	**0.74**	0.11
2. …is personally relevant	3.10 (1.16)	− 0.19	− 0.89		–	0.57***	0.61***	0.08***	0.24***	**0.74**	0.00
3. …motivates me to reduce the amount of alcohol I drink	3.23 (1.05)	− 0.25	− 0.58			–	0.61***	0.14***	0.33***	**0.76**	0.11
4. …makes me feel concerned about my drinking	2.86 (1.16)	0.07	− 0.92				–	− 0.03	0.16***	**0.84**	− 0.16
Factor 2—message perceptions											
5. …is easy to understand	4.26 (0.70)	− 1.09	2.80					–	0.43***	− 0.08	**0.66**
6. …is believable	3.93 (0.77)	− 1.02	2.14						–	0.16	**0.65**

Responses made on 5-point scales of 1 (*Strongly disagree*) to 5 (*Strongly agree*). Skew. = skewness; Kurt. = kurtosis.

***p < 0.001.

Bold values denote factor loadings > .40.

### Sample and procedure

Respondent recruitment was via an ISO-accredited web panel provider (Pureprofile). Eligible respondents were those aged 18–70 years (18 is the minimum legal alcohol purchase age in Australia) who consumed alcohol at least twice per month. Ethical approval was obtained from a university Human Research Ethics Committee and all participants provided informed consent. The study was conducted in accordance with relevant guidelines and regulations.

Although PME is widely used in campaign evaluation, a recent systematic review and meta-analysis noted that it has undergone little validation, especially using longitudinal approaches^5^. A longitudinal design was thus adopted to allow for the examination of predictive validity. Respondents completed measures at Time 1 (T1) and Time 2 (T2: three weeks after T1). The profiles of respondents at each time point are presented in Table 2. The sample was representative of the Australian drinking population in terms of gender and age^17^. The average age of those who dropped out was significantly lower than for those who remained in the sample at T2 (39.92 vs. 44.52; *p* < 0.001). There were no significant differences in attrition according to gender, socioeconomic status, or alcohol consumption.

**Table 2:** Sample profile and descriptive statistics for intentions and enactment (T1 and T2).

Characteristic	T1 *n* = 3001	T2 *n* = 1749
Gender		
Women (%)	51	51
Age (in years)	42.60 (*SD* = 14.88)	44.52 (*SD* = 15.04)
Socio-economic status^a^		
Low (%)	33	32
Mid (%)	44	44
High (%)	24	24
Alcohol consumption		
Drinks per week	7.82 (*SD* = 11.14)	8.03 (*SD* = 11.12)
> 4 drinks in a single sitting in previous 3 weeks (%)	55	44
Intentions^b^		
Count drinks	3.73 (1.22)	3.76 (1.22)
Decide not to exceed a certain number of drinks	3.84 (1.16)	3.87 (1.17)
Refuse a drink that is offered	3.65 (1.17)	3.64 (1.17)
Enactment^c^		
Counted drinks	3.42 (1.40)	3.44 (1.41)
Decided not to exceed a certain number of drinks	3.49 (1.34)	3.51 (1.35)
Refused a drink that was offered	2.78 (1.25)	2.73 (1.24)

Enactment intentions were measured on a scale of 1 (*Very unlikely*) to 5 (*Very likely*). A *Do not intend to drink alcohol* option was also provided (treated listwise). Enactment was measured on a scale of 1 (*Never*) to 5 (*Always*). A *Not applicable* option was also provided (treated listwise). T1 = Time 1. T2 = Time 2.

^a^As per Australian Bureau of Statistics’ Index of Relative Disadvantage^26^.

^b^*Do not intend to drink alcohol* responses—T1: *n* = 145, T2: *n* = 122.

^c^*Not applicable* responses—T1: *n* = 461, T2: *n* = 310.

### Measures

At T1, respondents were randomly assigned to view one of the following three messages promoting engagement in a PBS: ‘Keep count of your drinks’, ‘Decide how many drinks and stick to it’, and ‘It’s ok to say no if you’re offered a drink’. These messages were presented in multiple formats (see Supplementary Fig. S1 for examples featuring the ‘Keep count of your drinks’ message). The same PBS message was shown to respondents at both time points, along with items assessing the frequency with which respondents had engaged in the PBS during the past three weeks (1 = *Never* to 5 = *Always*; 6 = *Not applicable*) and how likely they were to engage over the next three weeks (1 = *Very unlikely* to 5 = *Very likely*; 6 = *Do not intend to drink alcohol*). *Not applicable* responses (T1: *n* = 461; T2: *n* = 310) and *Do not intend to drink alcohol* responses (T1: *n* = 145) were treated listwise. Respondents were also administered the AMPES, with responses to items made on a scale that ranged from 1 (*Strongly disagree*) to 5 (*Strongly agree*).

### Statistical analyses

An exploratory factor analysis (EFA) with direct oblimin rotation was performed on the AMPES scores from those assessed at T1 (*n* = 3001). A 95% parallel analysis was conducted to determine the number of factors to retain. In terms of item retention, those with a communality and factor loading > 0.50 were retained^20^.

McDonald’s omega and Cronbach’s alpha were calculated to assess internal consistency of scores on the items loading onto each factor. As a test of criterion validity, linear regression analyses were conducted to assess the relationships between each of the AMPES factors and respondents’ intentions to enact the PBSs, while controlling for prior PBS enactment. Regressions were also conducted to assess criterion validity of each AMPES item.

Data from those who provided information at both time points (*n* = 1749) were used to assess test re-test reliability and predictive validity. To determine test re-test reliability, the intraclass correlation coefficients (ICC) between T1 and T2 scores on each of the AMPES factors were calculated using two-way mixed effects models (type = absolute agreement)^21^. An ICC < 0.5 indicates poor reliability, values between 0.50 and 0.75 moderate reliability, values between 0.75 and 0.90 good reliability, and > 0.90 excellent reliability^21^.

To determine predictive validity, linear regressions were conducted to assess the relationships between each of the AMPES factors and the frequency with which respondents reported enacting the PBSs at T2, while controlling for prior enactment. Regressions were also conducted to assess the validity of each AMPES item. The Benjamini–Hochberg procedure was used to control the false discovery rate.

Analyses were conducted at both the overall and individual message level, with results from the latter presented in the online supplementary material. All analyses were conducted in SPSS v26.

## Results

### Exploratory factor analysis

Preliminary analyses indicated the data were acceptable for factor analytic examination (Bartlett’s test of sphericity: χ^2^(15) = 6243.48, *p* < 0.001; Kaiser–Meyer–Olkin index = 0.80). Item-level descriptive statistics and inter-item correlations are presented in Table 1 (for results relating to individual messages, see Tables S1 to S3 in the online supplementary material).

The EFA identified two factors underlying the AMPES. Factor 1 accounted for 49% of the variance in the data. This factor comprised four items primarily related to perceptions of effects (e.g., “this alcohol message makes me stop and think”; factor loadings: 0.74–0.84). Factor 2 accounted for a further 22% of variance, with two contributing items primarily related to message perceptions (e.g., “this alcohol message is understandable”; factor loadings: 0.65–0.66). Cross-loadings were not observed. The items loading onto each factor are presented in Table 1 (for results relating to individual messages, see Tables S1 to S3 in the online supplementary material).

### Internal consistency and test re-test reliability

The McDonald’s omega associated with scores on the items comprising Factor 1 was 0.85. McDonald’s omega for scores on the items comprising Factor 2 could not be calculated as this factor featured just two items. Accordingly, Cronbach’s alpha was calculated instead, with this found to be 0.60. The McDonald’s omega associated with scores on the full scale was 0.83. These coefficients suggest good internal consistency for scores on Factor 1 and the full scale, but suboptimal internal consistency for scores on Factor 2. For internal consistency results at the individual message level, see Tables S1 to S3 in the online supplementary material.

The ICC between Factor 1 T1 and T2 scores was 0.83 (95% CI 0.82, 0.85), indicating good test re-test reliability, while the ICC between Factor 2 T1 and T2 scores was 0.66 (95% CI 0.63, 0.69), indicating moderate reliability. The ICC for scores on the full scale was 0.80 (95% CI 0.78, 0.82). Table S4 in the online supplementary material provides the test re-test reliability results at the individual message level.

### Validity

Scores on both factors and the full scale demonstrated positive, moderate correlations with T1 enactment intentions (Factor 1: β_standardized_ = 0.23; Factor 2: β_standardized_ = 0.17; Full scale: β_standardized_ = 0.26; all *p*-values < 0.001), indicating good criterion validity. Additionally, T1 scores on each of the factors and the full scale significantly predicted T2 enactment frequency (Factor 1: β_standardized_ = 0.17, *p* < 0.001; Factor 2: β_standardized_ = 0.07, *p* = 0.001; Full scale: β_standardized_ = 0.18, *p* < 0.001), indicating adequate predictive validity. When Factors 1 and 2 were entered into the models simultaneously, Factor 2 remained correlated with T1 enactment intentions but was no longer found to be a significant predictor of T2 enactment. Full results from these analyses are presented in Table 3 (for results relating to individual messages, see Tables S5 to S7 in the online supplementary material).

**Table 3:** Regression analyses assessing the relationship between AMPES factors and (i) T1 PBS enactment intentions and (ii) T2 PBS enactment.

IV	T1 Intentions					T2 Enactment				
	B	SE	β	*p*	95% CI	B	SE	β	*p*	95% CI
T1 enactment	0.54	0.01	0.60	< 0.001	0.52, 0.56	0.52	0.02	0.49	< 0.001	0.48, 0.57
Factor 1	0.25	0.02	0.23	< 0.001	0.22, 0.28	0.22	0.03	0.17	< 0.001	0.16, 0.27

T1 enactment	0.53	0.01	0.59	< 0.001	0.51, 0.56	0.53	0.02	0.49	< 0.001	0.48, 0.57
Factor 2	0.28	0.02	0.17	< 0.001	0.24, 0.33	0.14	0.04	0.07	0.001	0.05, 0.22

T1 enactment	0.52	0.01	0.58	< 0.001	0.49, 0.54	0.51	0.02	0.48	< 0.001	0.47, 0.56
Factor 1	0.22	0.02	0.21	< 0.001	0.19, 0.25	0.21	0.03	0.16	< 0.001	0.15, 0.26
Factor 2	0.21	0.02	0.13	< 0.001	0.16, 0.25	0.07	0.04	0.04	0.110	− 0.02, 0.15

T1 enactment	0.53	0.01	0.58	< 0.001	0.50, 0.55	0.51	0.02	0.48	< 0.001	0.47, 0.56
Full scale	0.37	0.02	0.26	< 0.001	0.33, 0.41	0.29	0.04	0.18	< 0.001	0.22, 0.36

Enactment intentions were measured on a scale of 1 (*Very unlikely*) to 5 (*Very likely*). A *Do not intend to drink alcohol* option was also provided (treated listwise). Enactment was measured on a scale of 1 (*Never*) to 5 (*Always*). A *Not applicable* option was also provided (treated listwise). T1 = Time 1. T2 = Time 2.

Results relating to individual items of the AMPES are presented in Table 4. All items were found to be significantly associated with enactment intentions. With the exception of ‘easy to understand’, all items were found to predict T2 enactment (for results relating to individual messages, see Tables S8 to S10 in the online supplementary material).

**Table 4:** Regression analyses assessing the relationship between AMPES items and (i) T1 PBS enactment intentions and (ii) T2 PBS enactment.

IV	T1 Intentions					T2 Enactment				
	B	SE	β	*p*	95% CI	B	SE	β	*p*	95% CI
T1 enactment	0.54	0.01	0.60	< 0.001	0.51, 0.56	0.52	0.02	0.49	< 0.001	0.48, 0.57
Makes me stop and think about my drinking	0.20	0.01	0.21	< 0.001	0.17, 0.22	0.17	0.02	0.16	< 0.001	0.12, 0.21

T1 enactment	0.56	0.01	0.62	< 0.001	0.53, 0.58	0.54	0.02	0.51	< 0.001	0.49, 0.58
Is personally relevant	0.14	0.01	0.17	< 0.001	0.12, 0.17	0.10	0.02	0.10	< 0.001	0.06, 0.14

T1 enactment	0.52	0.01	0.58	< 0.001	0.50, 0.55	0.50	0.02	0.47	< 0.001	0.46, 0.55
Motivates me to reduce the amount of alcohol I drink	0.24	0.01	0.26	< 0.001	0.22, 0.27	0.22	0.02	0.20	< 0.001	0.17, 0.26

T1 enactment	0.56	0.01	0.62	< 0.001	0.54, 0.59	0.54	0.02	0.51	< 0.001	0.50, 0.58
Makes me feel concerned about my drinking	0.13	0.01	0.15	< 0.001	0.11, 0.15	0.13	0.02	0.13	< 0.001	0.09, 0.17

T1 enactment	0.55	0.01	0.61	< 0.001	0.52, 0.58	0.54	0.02	0.51	< 0.001	0.49, 0.58
Is easy to understand	0.18	0.02	0.12	< 0.001	0.14, 0.22	0.06	0.04	0.03	0.137	− 0.02, 0.13

T1 enactment	0.54	0.01	0.60	< 0.001	0.51, 0.56	0.52	0.02	0.49	< 0.001	0.48, 0.57
Is believable	0.21	0.02	0.17	< 0.001	0.18, 0.25	0.12	0.03	0.08	< 0.001	0.06, 0.18

Enactment intentions were measured on a scale of 1 (*Very unlikely*) to 5 (*Very likely*). A *Do not intend to drink alcohol* option was also provided (treated listwise). Enactment was measured on a scale of 1 (*Never*) to 5 (*Always*). A *Not applicable* option was also provided (treated listwise). T1 = Time 1. T2 = Time 2.

## Discussion

The present study aimed to develop and psychometrically evaluate a measure of PME (the AMPES) for messages promoting engagement in PBSs. Scale development was informed by recommendations from comprehensive systematic reviews of the PME literature, with the AMPES featuring items that (i) assessed a variety of persuasive constructs, (ii) referred to the behavior being targeted (alcohol consumption), and (iii) included a referent to improve measurement precision. Furthermore, a longitudinal design was adopted to allow for the examination of predictive validity.

Two distinct factors were found to underlie the AMPES: one related to ‘effects perceptions’ (Factor 1) and one to ‘message perceptions’ (Factor 2). Scores on the overall scale and the ‘effects perceptions’ factor demonstrated good internal consistency, whereas the internal consistency of scores on the ‘message perceptions’ factor was suboptimal. This suboptimal internal consistency is likely to be at least partially due to Factor 2 only comprising two items: Cronbach’s alpha is a function of the number of items in a scale and increases when a greater number of items is used to measure a construct^22^. Accordingly, a scale with a small number of items is less likely to produce a high alpha, regardless of how well items in that scale are correlated. The moderate correlation observed between the two items comprising Factor 2 (0.43) suggests the suboptimal Cronbach’s alpha may not be a reflection of the instability of this factor; however, caution should be exercised nevertheless. Given the items comprising Factor 2 tap into different elements of message perceptions (‘easy to understand’ relates to comprehension while ‘is believable’ relates to receptivity^6^), it may be more appropriate to consider the items comprising this factor individually when assessing PME.

Test re-test reliability, criterion validity, and predictive validity of scores on the overall scale and each of the factors were moderate to good. When comparing between factors, the effect sizes associated with relationships involving scores on the ‘effects perceptions’ factor were typically larger than those involving scores on the ‘message perceptions’ factor. These results likely reflect the goal-directed nature of human behavior, whereby engaging in a health-promoting activity is reliant on individuals expecting enactment to help them achieve their desired goal^23^. Understanding the utility of both effects and message perception measures has been cited as an important direction for future PME research^6^. Results of the present study thus represent an important contribution to the PME literature and suggest that measures assessing effects perceptions may be more valid predictors of actual effectiveness than those assessing message perceptions. These results also support the utility of an effects-based conceptualization of PME that focuses on the extent to which the target audience believes a message is likely to result in an effect that meets the objectives of the message (e.g., reduce alcohol consumption)^6^.

## Implications

The favorable results for scores on the AMPES provide evidence of the utility of the scale as a means of assessing perceived effectiveness of messages promoting engagement in PBSs. This constitutes a useful addition to the alcohol literature by facilitating rigorous assessments of the potential effectiveness of alcohol harm-minimization messages in a manner consistent with approaches used in the tobacco control field^5,10,19^. In research contexts where multiple questions are being posed and a short, validated scale is needed to minimize response burden, use of only the items comprising Factor 1 may be a preferred and sufficient test of PME given (i) the superior psychometric performance of the ‘effects perceptions’ factor relative to the ‘messages perceptions’ factor and (ii) the finding that the Factor 2 item ‘easy to understand’ failed to significantly predict enactment. The use of items that relate only to ‘effects perceptions’ has support in the literature, with some researchers recommending the development, testing, and validation of PME scales focusing only on effects^6,7^.

## Limitations and strengths

This study had several limitations. First, a web panel provider was used to recruit survey respondents. Future research could include samples generated via other means. Second, a confirmatory factor analysis was not conducted as only two items loaded onto Factor 2, making the model prone to estimation problems and specification errors^24^. Third, to ensure the AMPES was tested on a sample for whom alcohol messages are likely to be relevant, only drinkers (defined as those consuming alcohol at least twice per month) were surveyed. As such, the sample cannot be considered representative of all Australians.

Finally, the small to moderate magnitude of the validity correlations is consistent with the effect sizes observed in previous research assessing engagement in PBSs following the delivery of a message promoting their use^25^. This may reflect (i) the small number of message exposures (*n* = 2) and (ii) the modest 3-week timeframe between measurement points, which may not have been adequate to capture a sufficient number of drinking episodes. The ongoing debate about the utility of PME is a further consideration. While some have described PME as a valid yet imperfect measure of AME^3^, others argue that message recipients may not be able to accurately judge the persuasiveness of a message and that measures of actual effectiveness should thus be favored over measures of perceived effectiveness^9^. Such measures can, however, be difficult and costly to obtain^8^, making it important to produce robust measures of PME.

This study had several strengths. These included the (i) longitudinal design, which facilitated an assessment of predictive validity, (ii) development of the instrument based on recommendations from comprehensive systematic reviews on PME^5,6^, (iii) validation of the instrument in a non-college student sample, and (iv) use of a sample of drinkers (and thus respondents for whom the messages were likely to be relevant).

## Conclusion

The reliability and validity of the scores generated in this study indicate the likely usefulness of the proposed AMPES, especially those items related to effects perceptions. The AMPES provides intervention developers working in alcohol control with a validated tool to identify the likely effectiveness of PBS-promoting messages, thus assisting the development and evaluation of interventions designed to increase drinkers’ use of protective behavioral strategies. The ability of the AMPES to assess drinkers’ perceptions of the persuasiveness of PBS messages can assist in maximizing intervention effectiveness, thereby optimizing campaign expenditure. Assessing the applicability of the AMPES to other alcohol harm-minimization messages is warranted.

## Supplementary Information

Supplementary Information.

## Data Availability

To protect the privacy of individuals that participated in the study, the data underlying this article cannot be shared publicly*.* Please contact the corresponding author to request data from this study.
