# Supplementary material for: Development and validation of the Alcohol Message Perceived Effectiveness Scale
Source: Sci Rep. 2023 Jan 18;13:997. doi: 10.1038/s41598-023-28141-x (PMC9849458; doi:10.1038/s41598-023-28141-x)
Supplement: Supplementary file 1 — Supplementary Information. [file 41598_2023_28141_MOESM1_ESM.docx]

**Development and Validation of the Alcohol Message Perceived Effectiveness Scale**

**Supplementary Materials**

*Figure S1*

Drink counting message as presented to respondents


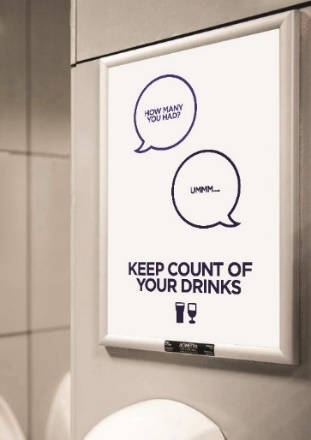

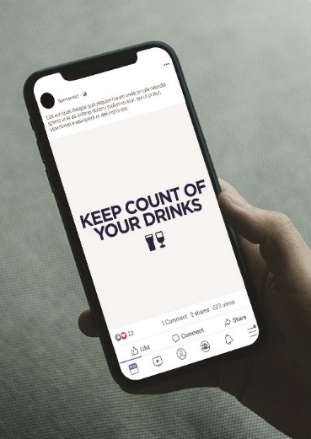

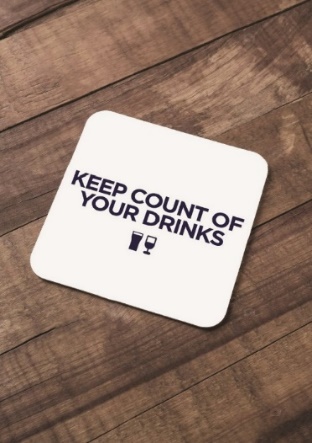


*Note*. In each of the survey waves, respondents were exposed to a protective behavioral strategy message in the formats shown above (i.e., each respondent saw a protective behavioral strategy message depicted in a series of three images).

*Table S1*

Item-level descriptive statistics, inter-item correlations, and factor loadings: ‘Keep count of your drinks’ (*n* = 1,001)

| Please indicate the extent to which you agree/disagree that this alcohol message… | Mean (*SD*) | Skew. | Kurt. | 1 | 2 | 3 | 4 | 5 | 6 | Loadings  (1) | Loadings  (2) |
| --- | --- | --- | --- | --- | --- | --- | --- | --- | --- | --- | --- |
| Factor 1  (50% of variance; McDonald’s omega = 0.85) |  |  |  |  |  |  |  |  |  |  |  |
| 1. …makes me stop and think about my drinking | 3.31 (1.07) | -0.39 | -0.61 | - | .54*** | .62*** | .58*** | .19*** | .36*** | **0.71** | 0.16 |
| 2. …is personally relevant | 3.10 (1.17) | -0.19 | -0.94 |  | - | .56*** | .60*** | .12*** | .25*** | **0.71** | 0.02 |
| 3. …motivates me to reduce the amount of alcohol I drink | 3.21 (1.06) | -0.18 | -0.68 |  |  | - | .63*** | .15*** | .34*** | **0.76** | 0.09 |
| 4. …makes me feel concerned about my drinking | 2.92 (1.17) | 0.06 | -0.95 |  |  |  | - | -.04 | .20*** | **0.88** | -0.19 |
| Factor 2  (22% of variance; Cronbach’s alpha = 0.63) |  |  |  |  |  |  |  |  |  |  |  |
| 5. …is easy to understand | 4.22 (0.70) | -1.12 | 3.01 |  |  |  |  | - | .47*** | -0.09 | **0.74** |
| 6. …is believable | 3.88 (0.80) | -0.97 | 1.85 |  |  |  |  |  | - | 0.18 | **0.63** |

*Note*. Responses made on 5-point scales of 1 (*Strongly disagree*) to 5 (*Strongly agree*). Skew. = skewness; Kurt. = kurtosis.

****p* < .001.

*Table S2*

Item-level descriptive statistics, inter-item correlations, and factor loadings: ‘Decide how many drinks and stick to it’ (*n* = 1,000)

| Please indicate the extent to which you agree/disagree that this alcohol message… | Mean (*SD*) | Skew. | Kurt. | 1 | 2 | 3 | 4 | 5 | 6 | Loadings  (1) | Loadings  (2) |
| --- | --- | --- | --- | --- | --- | --- | --- | --- | --- | --- | --- |
| Factor 1  (51% of variance; McDonald’s omega = 0.86) |  |  |  |  |  |  |  |  |  |  |  |
| 1. …makes me stop and think about my drinking | 3.31 (1.07) | -0.42 | -0.49 | - | .58*** | .67*** | .58*** | .16*** | .35*** | **0.74** | 0.12 |
| 2. …is personally relevant | 3.12 (1.16) | -0.24 | -0.83 |  | - | .60*** | .62*** | .09** | .30*** | **0.77** | -0.01 |
| 3. …motivates me to reduce the amount of alcohol I drink | 3.26 (1.04) | -0.34 | -0.46 |  |  | - | .60*** | .20*** | .40*** | **0.75** | 0.18 |
| 4. …makes me feel concerned about my drinking | 2.86 (1.14) | -0.02 | -0.92 |  |  |  | - | .01 | .19*** | **0.84** | -0.17 |
| Factor 2  (21% of variance; Cronbach’s alpha = 0.59) |  |  |  |  |  |  |  |  |  |  |  |
| 5. …is easy to understand | 4.23 (0.69) | -1.04 | 2.66 |  |  |  |  | - | .42*** | -0.07 | **0.64** |
| 6. …is believable | 3.86 (0.79) | -1.02 | 1.93 |  |  |  |  |  | - | 0.18 | **0.64** |

*Note*. Responses made on 5-point scales of 1 (*Strongly disagree*) to 5 (*Strongly agree*). Skew. = skewness; Kurt. = kurtosis.

***p* < .01. ****p* < .001.

*Table S3*

Item-level descriptive statistics, inter-item correlations, and factor loadings: ‘It’s ok to say no if you’re offered a drink’ (*n* = 1,000)

| Please indicate the extent to which you agree/disagree that this alcohol message… | Mean (*SD*) | Skew. | Kurt. | 1 | 2 | 3 | 4 | 5 | 6 | Loadings  (1) | Loadings  (2) |
| --- | --- | --- | --- | --- | --- | --- | --- | --- | --- | --- | --- |
| Factor 1  (48% of variance; McDonald’s omega = 0.85) |  |  |  |  |  |  |  |  |  |  |  |
| 1. …makes me stop and think about my drinking | 3.21 (1.08) | -0.32 | -0.66 | - | .56*** | .62*** | .60*** | .04 | .25*** | **0.76** | 0.07 |
| 2. …is personally relevant | 3.07 (1.15) | -0.16 | -0.88 |  | - | .56*** | .60*** | .02 | .18*** | **0.73** | -0.00 |
| 3. …motivates me to reduce the amount of alcohol I drink | 3.21 (1.04) | -0.24 | -0.58 |  |  | - | .61*** | .07* | .24*** | **0.76** | 0.08 |
| 4. …makes me feel concerned about my drinking | 2.80 (1.16) | 0.16 | -0.89 |  |  |  | - | -.05 | .10*** | **0.82** | -0.13 |
| Factor 2  (23% of variance; Cronbach’s alpha = 0.56) |  |  |  |  |  |  |  |  |  |  |  |
| 5. …is easy to understand | 4.32 (0.69) | -1.14 | 2.83 |  |  |  |  | - | .39*** | -0.07 | **0.58** |
| 6. …is believable | 4.04 (0.72) | -1.03 | 2.73 |  |  |  |  |  | - | 0.13 | **0.69** |

*Note*. Responses made on 5-point scales of 1 (*Strongly disagree*) to 5 (*Strongly agree*). Skew. = skewness; Kurt. = kurtosis.

**p* < .05. ****p* < .001.

*Table S4*

Test re-test reliability of scores on the AMPES (intraclass correlation coefficients), stratified by message

| Message | Factor 1 (T2)  ICC (95% CI) | Factor 2 (T2)  ICC (95% CI) | Full scale (T2)  ICC (95% CI) |
| --- | --- | --- | --- |
| Keep count of your drinks |  |  |  |
| Factor 1 (T1) | 0.83 (0.80, 0.86) | - | - |
| Factor 2 (T1) | - | 0.68 (0.62, 0.73) | - |
| Full scale (T1) | - | - | 0.81 (0.78, 0.84) |
| Decide how many drinks and stick to it |  |  |  |
| Factor 1 (T1) | 0.84 (0.81, 0.86) | - | - |
| Factor 2 (T1) | - | 0.69 (0.63, 0.73) | - |
| Full scale (T1) | - | - | 0.82 (0.79, 0.85) |
| It’s ok to say no if you’re offered a drink |  |  |  |
| Factor 1 (T1) | 0.83 (0.79, 0.85) | - | - |
| Factor 2 (T1) | - | 0.59 (0.52, 0.66) | - |
| Full scale (T1) | - | - | 0.78 (0.74, 0.81) |

*Note*. T1 = Time 1. T2 = Time 2. ICC = intraclass correlation coefficient.

*Table S5*

Regression analyses assessing the relationship between AMPES factors and (i) T1 PBS enactment intentions and (ii) T2 PBS enactment for the message ‘Keep count of your drinks’

|  | T1 Intentions | | | | | T2 Enactment | | | | |
| --- | --- | --- | --- | --- | --- | --- | --- | --- | --- | --- |
| IV | B | SE | β | *p* | 95% CI | B | SE | β | *p* | 95% CI |
| T1 enactment  Factor 1 | 0.52  0.27 | 0.02  0.03 | .58  .25 | <.001  <.001 | 0.47, 0.56  0.22, 0.32 | 0.56  0.13 | 0.04  0.05 | .52  .10 | <.001  .007 | 0.49, 0.64  0.04, 0.22 |
| T1 enactment  Factor 2 | 0.52  0.26 | 0.02  0.04 | .58  .16 | <.001  <.001 | 0.47, 0.56  0.18, 0.34 | 0.57  0.04 | 0.04  0.07 | .53  .02 | <.001  .532 | 0.50 0.65  -0.09, 0.18 |
| T1 enactment  Factor 1  Factor 2 | 0.50  0.24  0.16 | 0.02  0.03  0.04 | .56  .22  .11 | <.001  <.001  <.001 | 0.46, 0.54  0.19, 0.29  0.09, 0.24 | 0.56  0.13  -0.01 | 0.04  0.05  0.07 | .52  .10  -.01 | <.001  .008  .901 | 0.48, 0.64  0.03, 0.22  -0.15, 0.13 |
| T1 enactment  Full scale | 0.50  0.38 | 0.02  0.03 | .56  .27 | <.001  <.001 | 0.46, 0.55  0.31, 0.45 | 0.56  0.16 | 0.04  0.06 | .52  .09 | <.001  .010 | 0.48, 0.63  0.04, 0.28 |

*Note*. Enactment intentions were measured on a scale of 1 (*Very unlikely*) to 5 (*Very likely*). A *Do not intend to drink alcohol* option was also provided (treated listwise). Enactment was measured on a scale of 1 (*Never*) to 5 (*Always*). A *Not applicable* option was also provided (treated listwise). T1 = Time 1. T2 = Time 2.

*Table S6*

Regression analyses assessing the relationship between AMPES factors and (i) T1 PBS enactment intentions and (ii) T2 PBS enactment for the message ‘Decide how many drinks and stick to it’

|  | T1 Intentions | | | | | | T2 Enactment | | | | |
| --- | --- | --- | --- | --- | --- | --- | --- | --- | --- | --- | --- |
| IV | B | SE | β | *p* | 95% CI | B | | SE | β | *p* | 95% CI |
| T1 enactment  Factor 1 | 0.53  0.27 | 0.02  0.03 | .59  .25 | <.001  <.001 | 0.49, 0.57  0.22, 0.32 | 0.47  0.30 | | 0.04  0.05 | .44  .24 | <.001  <.001 | 0.39, 0.54  0.21, 0.40 |
| T1 enactment  Factor 2 | 0.52  0.32 | 0.02  0.04 | .58  .20 | <.001  <.001 | 0.47, 0.56  0.24, 0.39 | 0.45  0.26 | | 0.04  0.08 | .43  .14 | <.001  <.001 | 0.37, 0.53  0.12, 0.41 |
| T1 enactment  Factor 1  Factor 2 | 0.51  0.23  0.22 | 0.02  0.03  0.04 | .57  .21  .14 | <.001  <.001  <.001 | 0.47, 0.55  0.17, 0.28  0.14, 0.30 | 0.44  0.28  0.15 | | 0.04  0.05  0.08 | .42  .22  .08 | <.001  <.001  .040 | 0.37, 0.52  0.19, 0.37  0.01, 0.30 |
| T1 enactment  Full scale | 0.52  0.39 | 0.02  0.03 | .58  .28 | <.001  <.001 | 0.47, 0.56  0.32, 0.45 | 0.44  0.43 | | 0.04  0.06 | .42  .25 | <.001  <.001 | 0.37, 0.52  0.31, 0.55 |

*Note*. Enactment intentions were measured on a scale of 1 (*Very unlikely*) to 5 (*Very likely*). A *Do not intend to drink alcohol* option was also provided (treated listwise). Enactment was measured on a scale of 1 (*Never*) to 5 (*Always*). A *Not applicable* option was also provided (treated listwise). T1 = Time 1. T2 = Time 2.

*Table S7*

Hierarchical regression analyses assessing the relationship between AMPES factors and (i) T1 PBS enactment intentions and (ii) T2 PBS enactment for the message: ‘It’s ok to say no if you’re offered a drink’

|  | T1 Intentions | | | | | | T2 Enactment | | | | |
| --- | --- | --- | --- | --- | --- | --- | --- | --- | --- | --- | --- |
| IV | B | SE | β | *p* | 95% CI | B | | SE | β | *p* | 95% CI |
| T1 enactment  Factor 1 | 0.57  0.22 | 0.02  0.03 | .63  .21 | <.001  <.001 | 0.53, 0.62  0.17, 0.27 | 0.55  0.22 | | 0.04  0.05 | .51  .18 | <.001  <.001 | 0.47, 0.62  0.13, 0.31 |
| T1 enactment  Factor 2 | 0.57  0.27 | 0.02  0.04 | .62  .16 | <.001  <.001 | 0.53, 0.61  0.19, 0.35 | 0.55  0.11 | | 0.04  0.07 | .51  .06 | <.001  .143 | 0.47, 0.63  -0.04, 0.26 |
| T1 enactment  Factor 1  Factor 2 | 0.55  0.20  0.23 | 0.02  0.03  0.04 | .61  .19  .13 | <.001  <.001  <.001 | 0.51, 0.60  0.15, 0.25  0.15, 0.31 | 0.54  0.22  0.07 | | 0.04  0.05  0.07 | .50  .18  .04 | <.001  <.001  .355 | 0.46, 0.62  0.13, 0.31  -0.08, 0.21 |
| T1 enactment  Full scale | 0.56  0.35 | 0.02  0.04 | .61  .24 | <.001  <.001 | 0.52, 0.60  0.28, 0.42 | 0.54  0.31 | | 0.04  0.06 | .50  .19 | <.001  <.001 | 0.46, 0.61  0.19, 0.43 |

*Note*. Enactment intentions were measured on a scale of 1 (*Very unlikely*) to 5 (*Very likely*). A *Do not intend to drink alcohol* option was also provided (treated listwise). Enactment was measured on a scale of 1 (*Never*) to 5 (*Always*). A *Not applicable* option was also provided (treated listwise). T1 = Time 1. T2 = Time 2.

*Table S8*

Regression analyses assessing the relationship between AMPES items and (i) T1 PBS enactment intentions and (ii) T2 PBS enactment for the message ‘Keep count of your drinks’

|  | | T1 Intentions | | | | | | T2 Enactment | | | | | |
| --- | --- | --- | --- | --- | --- | --- | --- | --- | --- | --- | --- | --- | --- |
| IV | B | | SE | β | *p* | 95% CI | B | | SE | β | *p* | 95% CI |  |
| T1 enactment  Makes me stop and think about my drinking | 0.52  0.23 | | 0.02  0.02 | .58  .25 | <.001  <.001 | 0.48, 0.56  0.18, 0.27 | 0.56  0.10 | | 0.04  0.04 | .52  .09 | <.001  .012 | 0.49, 0.64  0.02, 0.18 |  |
| T1 enactment  Is personally relevant | 0.54  0.13 | | 0.02  0.02 | .61  .16 | <.001  <.001 | 0.50, 0.58  0.09, 0.18 | 0.58  0.00 | | 0.04  0.04 | .54  .00 | <.001  .914 | 0.50, 0.65  -0.07, 0.08 |  |
| T1 enactment  Motivates me to reduce the amount of alcohol I drink | 0.50  0.26 | | 0.02  0.02 | .56  .28 | <.001  <.001 | 0.46, 0.54  0.21, 0.30 | 0.54  0.15 | | 0.04  0.04 | .51  .13 | <.001  <.001 | 0.47, 0.62  0.07, 0.23 |  |
| T1 enactment  Makes me feel concerned about my drinking | 0.54  0.13 | | 0.02  0.02 | .61  .16 | <.001  <.001 | 0.50, 0.58  0.09, 0.18 | 0.57  0.11 | | 0.04  0.04 | .53  .11 | <.001  .002 | 0.49, 0.64  0.04, 0.19 |  |
| T1 enactment  Is easy to understand | 0.53  0.18 | | 0.02  0.04 | .60  .13 | <.001  <.001 | 0.49, 0.58  0.11, 0.25 | 0.58  -0.01 | | 0.04  0.06 | .54  -.00 | <.001  .927 | 0.50, 0.66  -0.13, 0.12 |  |
| T1 enactment  Is believable | 0.52  0.19 | | 0.02  0.03 | .59  .15 | <.001  <.001 | 0.48, 0.57  0.12, 0.25 | 0.57  0.06 | | 0.04  0.06 | .53  .04 | <.001  .285 | 0.49, 0.65  -0.05, 0.17 |  |

*Note*. Enactment intentions were measured on a scale of 1 (*Very unlikely*) to 5 (*Very likely*). A *Do not intend to drink alcohol* option was also provided (treated listwise). Enactment was measured on a scale of 1 (*Never*) to 5 (*Always*). A *Not applicable* option was also provided (treated listwise). T1 = Time 1. T2 = Time 2.

*Table S9*

Regression analyses assessing the relationship between AMPES items and (i) T1 PBS enactment intentions and (ii) T2 PBS enactment for the message ‘Decide how many drinks and stick to it’

|  | T1 Intentions | | | | | | T2 Enactment | | | | |
| --- | --- | --- | --- | --- | --- | --- | --- | --- | --- | --- | --- |
| IV | B | SE | β | *p* | 95% CI | B | | SE | β | *p* | 95% CI |
| T1 enactment  Makes me stop and think about my drinking | 0.53  0.22 | 0.02  0.02 | .59  .23 | <.001  <.001 | 0.48, 0.57  0.17, 0.26 | 0.46  0.26 | | 0.04  0.04 | .44  .24 | <.001  <.001 | 0.39, 0.54  0.18, 0.34 |
| T1 enactment  Is personally relevant | 0.54  0.16 | 0.02  0.02 | .61  .18 | <.001  <.001 | 0.50, 0.59  0.12, 0.20 | 0.48  0.17 | | 0.04  0.04 | .46  .16 | <.001  <.001 | 0.40, 0.55  0.09, 0.24 |
| T1 enactment  Motivates me to reduce the amount of alcohol I drink | 0.52  0.26 | 0.02  0.02 | .58  .27 | <.001  <.001 | 0.48, 0.56  0.22, 0.31 | 0.44  0.30 | | 0.04  0.04 | .42  .27 | <.001  <.001 | 0.37, 0.52  0.22, 0.38 |
| T1 enactment  Makes me feel concerned about my drinking | 0.56  0.13 | 0.02  0.02 | .62  .15 | <.001  <.001 | 0.51, 0.60  0.09, 0.17 | 0.49  0.15 | | 0.04  0.04 | .47  .14 | <.001  <.001 | 0.41, 0.57  0.07, 0.22 |
| T1 enactment  Is easy to understand | 0.54  0.21 | 0.02  0.04 | .60  .15 | <.001  <.001 | 0.49, 0.58  0.14, 0.28 | 0.47  0.14 | | 0.04  0.07 | .45  .08 | <.001  .037 | 0.39, 0.55  0.01, 0.28 |
| T1 enactment  Is believable | 0.52  0.22 | 0.02  0.03 | .59  .18 | <.001  <.001 | 0.48, 0.57  0.16, 0.29 | 0.45  0.19 | | 0.04  0.06 | .43  .14 | <.001  <.001 | 0.37, 0.53  0.08, 0.30 |

*Note*. Enactment intentions were measured on a scale of 1 (*Very unlikely*) to 5 (*Very likely*). A *Do not intend to drink alcohol* option was also provided (treated listwise). Enactment was measured on a scale of 1 (*Never*) to 5 (*Always*). A *Not applicable* option was also provided (treated listwise). T1 = Time 1. T2 = Time 2.

*Table S10*

Regression analyses assessing the relationship between AMPES items and (i) T1 PBS enactment intentions and (ii) T2 PBS enactment for the message ‘It’s ok to say no if you’re offered a drink’

|  | T1 Intentions | | | | | | T2 Enactment | | | | |
| --- | --- | --- | --- | --- | --- | --- | --- | --- | --- | --- | --- |
| IV | B | SE | β | *p* | 95% CI | B | | SE | β | *p* | 95% CI |
| T1 enactment  Makes me stop and think about my drinking | 0.57  0.15 | 0.02  0.02 | .63  .17 | <.001  <.001 | 0.53, 0.62  0.11, 0.20 | 0.55  0.15 | | 0.04  0.04 | .51  .15 | <.001  <.001 | 0.47, 0.62  0.08, 0.23 |
| T1 enactment  Is personally relevant | 0.59  0.14 | 0.02  0.02 | .65  .16 | <.001  <.001 | 0.55, 0.63  0.10, 0.18 | 0.56  0.15 | | 0.04  0.04 | .52  .15 | <.001  <.001 | 0.48, 0.64  0.08, 0.22 |
| T1 enactment  Motivates me to reduce the amount of alcohol I drink | 0.56  0.21 | 0.02  0.02 | .61  .22 | <.001  <.001 | 0.51, 0.60  0.17, 0.26 | 0.53  0.20 | | 0.04  0.04 | .50  .19 | <.001  <.001 | 0.45, 0.61  0.12, 0.28 |
| T1 enactment  Makes me feel concerned about my drinking | 0.59  0.12 | 0.02  0.02 | .65  .14 | <.001  <.001 | 0.55, 0.63  0.08, 0.16 | 0.56  0.13 | | 0.04  0.04 | .52  .13 | <.001  <.001 | 0.48, 0.64  0.05, 0.20 |
| T1 enactment  Is easy to understand | 0.58  0.14 | 0.02  0.04 | .64  .10 | <.001  <.001 | 0.54, 0.63  0.07, 0.21 | 0.56  0.04 | | 0.04  0.07 | .52  .02 | <.001  .591 | 0.48, 0.64  -0.09, 0.16 |
| T1 enactment  Is believable | 0.57  0.23 | 0.02  0.03 | .63  .17 | <.001  <.001 | 0.53, 0.62  0.16, 0.30 | 0.55  0.10 | | 0.04  0.06 | .51  .07 | <.001  .073 | 0.47, 0.63  -0.01, 0.22 |

*Note*. Enactment intentions were measured on a scale of 1 (*Very unlikely*) to 5 (*Very likely*). A *Do not intend to drink alcohol* option was also provided (treated listwise). Enactment was measured on a scale of 1 (*Never*) to 5 (*Always*). A *Not applicable* option was also provided (treated listwise). T1 = Time 1. T2 = Time 2.
